# Dissection, Culture and Analysis of Primary Cranial Neural Crest Cells from Mouse for the Study of Neural Crest Cell Delamination and Migration

**DOI:** 10.3791/60051

**Published:** 2019-10-03

**Authors:** Sandra Guadalupe Gonzalez Malagon, Lisa Dobson, Anna M Lopez Muñoz, Marcus Dawson, William Barrell, Petros Marangos, Matthias Krause, Karen J Liu

**Affiliations:** 1 Centre for Craniofacial and Regenerative Biology, King's College London; 2 Institute of Molecular Biology and Biotechnology, FORTH, Department of Biomedical Research, University of Ioannina; 3 Randall Centre of Cell & Molecular Biophysics, King's College London; 4 Department of Biological Applications and Technology, University of Ioannina

**Keywords:** Developmental Biology, Issue 152, Cell culture, live imaging, mouse cranial neural crest, cell migration, epithelial-mesenchymal transition, cell motility

## Abstract

Over the past several decades there has been an increased availability of genetically modified mouse models used to mimic human pathologies. However, the ability to study cell movements and differentiation *in vivo* is still very difficult. Neurocristopathies, or disorders of the neural crest lineage, are particularly challenging to study due to a lack of accessibility of key embryonic stages and the difficulties in separating out the neural crest mesenchyme from adjacent mesodermal mesenchyme. Here, we set out to establish a well-defined, routine protocol for the culture of primary cranial neural crest cells. In our approach we dissect out the mouse neural plate border during the initial neural crest induction stage. The neural plate border region is explanted and cultured. The neural crest cells form in an epithelial sheet surrounding the neural plate border, and by 24 h after explant, begin to delaminate, undergoing an epithelial-mesenchymal transition (EMT) to become fully motile neural crest cells. Due to our two-dimensional culturing approach, the distinct tissue populations (neural plate versus premigratory and migratory neural crest) can be readily distinguished. Using live imaging approaches, we can then identify changes in neural crest induction, EMT and migratory behaviors. The combination of this technique with genetic mutants will be a very powerful approach for understanding normal and pathological neural crest cell biology.

## Introduction

The neural crest (NC) lineage is a transient, multipotent and migratory population of cells that appears exclusively in vertebrates during early embryonic development^1,2^. Neural crest derivatives are extremely diverse, and include glia, smooth muscle, melanocytes, neurons and craniofacial bone and cartilage^3,4^. Because the neural crest contributes to the function of many organ systems, this lineage is essential for human embryogenesis. Aberrant NC development is implicated in a wide range of the most common human birth defects (i.e., cleft lip and palate)^5^, and also disorders such as Hirschsprung’s disease (HSCR), Wardensburg syndrome (WS), CHARGE syndrome and Williams Syndrome^6,7,8,9^.

NC development has been explored in a number of non-mammalian model systems including *Xenopus*, chick and zebrafish models. In mammals, work in mouse models has identified some of the key genetic events underlying neural crest development; however, it has been more difficult to follow the cell biology of neural crest migration, due to the inaccessibility of the mouse embryo (reviewed elsewhere^10,11^). Furthermore, while studies in chick, *Xenopus* and zebrafish have established a gene regulatory network for NC, loss of function studies in these animal models sometimes do not exhibit a comparable phenotype in mouse. For example, in *Xenopu*s, zebrafish and chick, non-canonical Wnt signaling is one of the cellular mechanisms that allows the NC to acquire its migratory capacity^12,13,14,15^. However, in mouse, loss of non-canonical Wnt signaling does not seem to affect migration^16^. As *in vivo* NC migration has been difficult to track for long periods in mouse, it is unclear whether these species-differences reflect differing modes of migration, or differences in molecular regulation.

As noted, NC studies in mouse have been very challenging because the *ex utero* culture of embryos is laborious. Moreover, the NC is constantly in intimate contact with adjacent tissues such as mesoderm and neurectoderm. Recent use of neural crest-specific *Cre* drivers or exogenous dyes has allowed us to fluorescently label the migratory NC; however, these approaches are still limited. Despite multiple reports describing different techniques to visualize NC migration^17,18^, it has been difficult to resolve these techniques into a simple and routine procedure.

It is clear that there is a need for techniques that allow the handling and characterization of mammalian NC. We focused our efforts on the mouse cranial NC as it is the primary model for studying human craniofacial development and neurocristopathies. We refined our approach based on several interesting reports describing primary culture of NC cells^19,20,21^. Here, we thoroughly describe the optimal culture techniques for explanting primary NC cells. We demonstrate the live cell imaging method and the optimal use of different matrices to coat the culture plates. Our protocol describes how to capture the migration of live NC cells using an inverted microscope, which is intended as a guideline for use with other microscopes, as well as a detailed summary of our cellular analyses.

The expected result from the explant should be a beautifully laid out distribution of cells that are clearly distinguished under the microscope, where one can see three different populations of cells which represent (i) neural plate, (ii) premigratory, and, (iii) migratory neural crest cells. We demonstrate how to analyze the cell behaviors at the border of the premigratory population of cells during the epithelial-mesenchymal transition. We also focused our effort on studying fully migratory cells for cell speed, distance and cell morphology.

## Protocol

All animal work has undergone ethical approval by the King’s College London Ethical Review Process and was performed in accordance with UK Home Office Project License P8D5E2773 (KJL).

### 1 Preparation of reagents

1. Prepare general solutions and tools including sterile phosphate buffer saline (PBS), 70% ethanol, dissection tools (forceps and dissection blades or sterile needles), plastic plates or glass slides coated with a commercially available extracellular matrix (ECM)-based hydrogel or fibronectin (see the **Table of Materials**), and neural crest media (see below).
2. Prepare the neural crest basal medium using Dulbecco’s modified Eagle’s medium (DMEM, 4500 mg/L glucose), 15% fetal bovine serum (FBS), 0.1 mM minimum essential medium nonessential amino acids (MEM NEAA 100X), 1 mM sodium pyruvate, 55 μM β-mercaptoethanol, 100 units/mL penicillin, 100 units/mL streptomycin, and 2 mM L-glutamine.
  1. Condition the media overnight using growth-inhibited STO feeder cells^21^.
    1. Prepare STO cells (see the **Table of Materials**) media to contain DMEM supplemented by 10% FBS and 100 U/mL penicillin, 100 U/mL streptomycin. Grow and expand STO cells to confluence in 25 cm^2^ flasks coated with 0.1% gelatin. Apply 5000 rad of gamma irradiation.
    2. Seed approximately 3 x 10^6^ growth-inhibited cells onto a 10 cm^2^ dish or 25 cm^2^ flask (from step 1.2.1.1). Add approximately 10–12 mL of neural crest basal medium and incubate overnight.
      NOTE: Seeded cells can be used to produce conditional medium for up to 10 days. Check appearance of cells regularly
  2. Filter the medium (0.22 μm pore size), and supplement with 25 ng/mL basic fibroblast growth factor (bFGF) and 1000 U of leukemia inhibitor factor (LIF).
    NOTE: Store at 4 °C and use within a month or store at -20 °C and use within 3 months.
3. Coat the tissue culture surfaces with extracellular matrix.
  NOTE: Depending on the biological question being asked, the matrix can be coated onto glass-bottomed culture dishes, plastic tissue culture dishes or glass cover slips. See below for differing ECM based hydrogel dilutions dependent on the matrix substrate. Fibronectin has been tested on glass-bottomed dishes and cover slips only at the concentrations specified below. Here on, we will refer to the substrate-coated surfaces as “coated plates”.
  1. Coat the tissue culture surfaces with ECM based hydrogel.
    NOTE: Keep the substrate cold until plating, either by cooling the media or keeping on ice.
    1. Thaw the hydrogel at 4 °C overnight. Add 5 mL of 10% FBS in DMEM to 5 mL of hydrogel for a final volume of 10 mL (see the **Table of Materials**).
    2. Make 0.5–1 mL aliquots as convenient and store at -20 °C.
    3. Thaw the hydrogel aliquots on ice.
    4. Use a 1:20 dilution of the hydrogel stock to coat plastic.
    5. Use a 1:5 dilution of the hydrogel stock to coat glass slides and glass-bottomed tissue culture plates.
      NOTE: Dilute the hydrogel in **cold** DMEM.
    6. Apply enough diluted hydrogel to cover the desired area on to plates/slides and incubate for 30–45 min at 37 °C.
    7. Use coated plates/slides immediately or store coated slides at 4 °C overnight.
    8. Remove excesses and rinse slides with high glucose DMEM (optional) before use.
  2. Coat the tissue culture surfaces with fibronectin.
    1. Make aliquots of 1 mg/mL fibronectin stock solution and store at -80 °C. Dilute fibronectin with Dulbecco’s PBS (dPBS) to a final concentration of 1 μg/mL.
    2. Apply sufficient fibronectin to cover the desired area and incubate at room temperature for 15 min.
    3. Remove residual fibronectin and allow the glass to dry for 30-45 min.
    4. Rinse wells or cover slips with high glucose DMEM (optional) before use.

### 2 Day 1: Dissection of early somite stage embryos

NOTE: Use sterile tools and sterile solutions. If genotyping is needed, collect the body of the embryo for DNA extraction.

**Figure 1:**
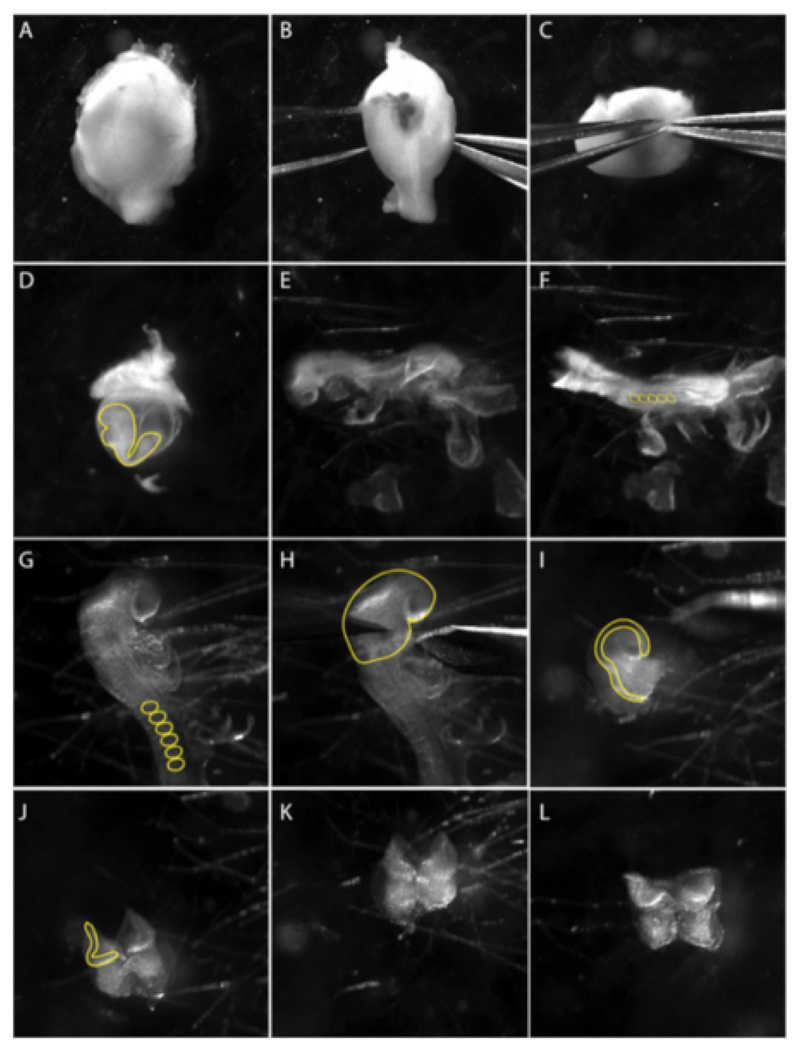
Isolation of cranial neural crest explants from an e8.5 embryo. Images are stills from a video documenting the micro-dissection technique. **(A–C)** Dissection of the embryo from the uterus. **(B–C)** Using two sharp forceps, gently pull apart the muscular layer. Panel **(D)** shows embryo inside the visceral yolk sac (yellow line). Extract the embryo from the visceral yolk sac. **(E)** Lateral views of the embryo at stage 8.5 lateral. **(F)** Dorsal view of the embryo at stage 8.5. Count somites (ss) to determine the age of embryos; usually 5–8 ss (yellow circles in F). **(G)** Close up look at the cranial region of the embryo. Remove extraembryonic membranes from the cranial region; somites are marked with a yellow line. **(H)** Dissections of anterior neural plate are performed under the first branchial arch (yellow line). **(I)** Lateral view of anterior neural plate dissection. Neural folds, where neural crest cells arise, are marked with a yellow line. **(J–L)** Remove mesodermal tissue (fluffy mesenchymal cells) underlying anterior neural folds as much as possible before plating NP onto prepared culture dishes. Movie was taken using a stereo-microscope with a widefield apochromatic lens at 3.0X zoom (see the **Table of Materials**).

**Figure 2:**
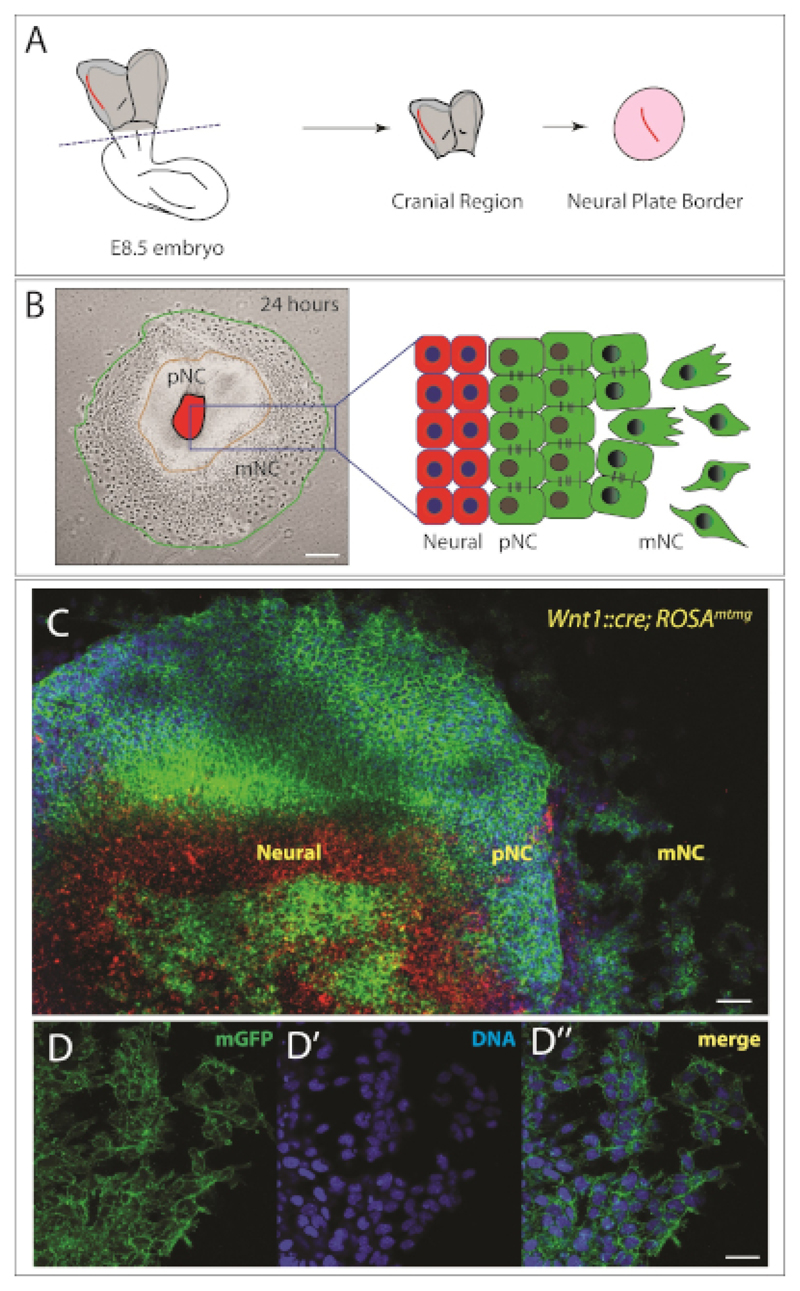
Murine cranial neural crest explant. **(A)** Schematic representation of the dorsal view of an e8.5 mouse embryo. The cranial region of the embryo is cut at the dashed line. The neural plate border (highlighted in red) is isolated from the surrounding mesoderm tissue and cultured for 24 h to allow the cranial neural crest to emigrate. Schematic adapted from^22,23^. **(B)** Left: Representative bright field image of a cranial neural crest explant 24 hours after plating. Three populations of cells are observed, which are also schematized on the right. NP = neural plate, pNC = pre-migratory neural crest and mNC = migratory neural crest. Scale bar = 250 μm. **(C)** Higher magnification images of an explant from genetically labeled mouse (*Wnt1::cre; Rosa^mTmG^*). Cells without the Cre driver express membrane tomato (mT) in red. Expression of Cre under the control of a neural crest specific Wnt1 promoter leads to excision of the mT cassette and expression of membrane GFP (mG) in green. Nuclei are stained with Hoescht (in blue). Scale bar = 200 μm. **(D–D”)** Higher magnification images of migratory cells expressing membrane GFP (D). **(D’)** DNA is labeled with Hoescht (blue). **(D”)** Merge of D and D’. Scale bar = 20 μm.

1. Dissection of the cranial neural plate is restricted to embryos at 8.5 days post coitum (dpc). Select embryos at the 5–8 somite stage. Dissect the uterus into PBS and cut the mesometrium to separate each embryo (Figure 1A). The muscular wall of the uterus contracts and the decidual tissue will become visible (Figure 1B).
  NOTE: Maintain embryos in the uterus in ice-cold PBS while dissections are performed one embryo at a time. Move embryos with a glass Pasteur pipette into fresh sterile PBS to improve visibility and reduce contamination.
2. Slide forceps between the muscle layer and decidual tissue and remove the muscle layer with a second pair of forceps (Figure 1C).
3. Using forceps, pierce the deciduum at the edges of the mesometrial pole and with a second pair of forceps tear to open perpendicularly to the pole.
4. Peel back the decidual tissue with the forceps to visualize the Reichert’s membrane.
5. Remove Reichert’s membrane carefully. The visceral yolk sac becomes visible and the embryo can be seen inside (Figure 1D).
6. Remove the visceral yolk sac and the amnion (Figure 1E) and position the embryo in order to visualize the head fold (Figure 1F).
7. Cut the head fold above the heart and scrape away the underlying mesoderm using forceps and/or eyelash tools to obtain a clean neural plate (NP) (Figure 1H).
  NOTE: The NP can be kept whole or divided down the anteroposterior axis so that each side can be plated individually. The neural plate border can be further trimmed away from the neural plate in order to minimize neuronal contributions to the explants.
8. Use a glass Pasteur pipette to transfer the dissected neural plate onto a hydrogel-coated dish filled with conditioned neural crest media.
9. Gently swirl the dish to position the NP in the middle of the well. This is important for maximizing the phase quality for live-cell imaging (on day 2).
10. Incubate overnight (or to desired time-point) at 37 °C in 5% CO_2_. Neural crest cells should be visibly migrating out of the neural plate.
  NOTE: Cells usually attach within 6–8 h. After the explant attaches, allow more time to visualize migrating cells. Usually by 24 h post explant, we can find three distinguishable populations of cells. The first population, at the center of the explant, is the neural plate (NP). The second population, the premigratory NC (pNC), surrounds the NP in an epithelial sheet of cells. The third population, in the outside ring, is formed of migratory NC (mNC), which are bigger in size, and appear fully mesenchymal (Figure 2).

### 3 Day 2: Live cell imaging of murine cranial neural crest cells

NOTE: Imaging should be performed at 24 h post explanting to optimally image and quantify neural crest cell migration. NC induction media does not need to be refreshed before live cell imaging. Access to an inverted microscope, with a motorized stage and an incorporated environment chamber is required. Use multi-well tissue culture dishes suitable for imaging (**Table of Materials**).

1. **Microscope set-up**
  1. Set the environment chamber at 37 °C and 5% CO_2_.
  2. Pierce a hole into the lid of the tissue culture plate lid to allow the CO_2_ needle, connected to the CO_2_ humidification chamber, to sit within the plate.
  3. Place the tissue culture dish into the specimen holder and tape down the plate lid and CO_2_ needle to prevent shaking during multi-well acquisition.
  4. Switch on the microscope controller, the stage controller and the imaging software.
  5. Focus on the cranial NC cells at 10x magnification (with matching phase ring in the condenser selected).
  6. Set high quality phase-contrast on the microscope by adjusting the field iris diaphragm, aperture iris diaphragm and centering telescope, as specified in the microscope set-up manual.
2. **Phase contrast live-cell imaging**
  1. Set the directory or file location where the time-lapse files will be saved.
  2. Set the exposure time, binning, and camera area.
  3. Set the number of time points, duration of imaging and time interval between frames.
    1. To quantify NC cell migratory capacity, set the microscope to 10x magnification, taking 1 frame every 5 min (217 time points over 18 h). To quantify cell morphology, set magnification to 40x, taking 1 frame/min (61 time points over 1 h). To quantify lamellipodial dynamics, set magnification to 40x or 60x magnification, taking 1 frame every 10 s (over 10 min).
  4. For multi-well imaging, set the mechanical stage to move between selected XY positions of interest. Confirm that the cranial NC cells are in focus and the stage positions are correct.
  5. Use the **Acquire** command to start time-lapse imaging.
  6. Once time-lapse imaging is complete, review the multidimensional data and export .stk files for analysis.
    NOTE: .stk is a TIFF stack file.
  7. Exit the software, shut down the computer and turn off the stage, camera and microscope controllers.

### 4 Imaging analysis: quantification of neural crest cell migration

NOTE: To better define the cellular behaviors exhibited by migrating murine cranial neural crest cells, we have analyzed a series of quantifiable migratory parameters, specifically focusing on migratory capacity and cell shape dynamics. (1) **Migration (accumulated distance)** is the total path length taken by the cell (μm); (2) **Migration (Euclidean distance)** is the straight-line distance between initial and final position of cell (μm); (3) **Migration (cell speed)** is distance traveled by cell per unit of time (μm/min); (4) **Cell Shape (cell area)** is the total surface covered by cell. Set pixel to micron scale according to imaging microscope. (A = A_px_ x N_px_, where A_px_ = pixel area and N_px_ = number of pixels. Units: μm^2^; (5) **Cell shape (cell circularity)** is the deviation of cell shape from a perfect circle which is indicated by a circularity value of 1.0 (4π (A/P^2^)) where A = area and P = perimeter.

1. **Single cell tracking**
  NOTE: To measure NC cell migration, XY coordinates of individual cells across all time-lapse frames are generated. This allows for subsequent analysis of distance, speed and persistence measures of cell migration.
  1. Open ImageJ and import data as TIFF stack files.
  2. Click **Analyze | Set Scale** to calibrate the .stk files according to microscope settings, working in pixel/μm.
  3. Click **Plugins | Tracking | Manual Tracking** to open Image J manual cell tracking plugin. To begin cell tracking, select **Add track**.
  4. Track cells through all frames of time-lapse movies, using the nucleus as a reference point.
    NOTE: 10–20 cells should be tracked per explant, with a total of 60 cells tracked (n = 3). Cells that undergo cell division during the course of time-lapse should be excluded from analysis.
  5. Save and export the results as a .csv file. Results represent individual cell track number, slice number and XY coordinates over all frames.
2. **Quantification of neural crest cell migratory capacity**
  1. Open single cell tracking data (see above). Convert .csv files into .txt file format.
  2. Open the migration software (the **Table of Materials**). Click the **Import Data** tab to import the cell tracking data as a .txt file.
  3. Under **Datasets | Initialization**, select the number of slices or frames to be analyzed and set the XY calibration and time interval between frames. Select **Apply settings** to save the settings.
  4. Select the **Plot Data** symbol to form trajectory plots. Select the **Statistics** symbol to quantify distance and speed measures.
  5. Save the trajectory plots as bitmap (.bmp) files, and distance and speed measures as .txt files. Select the **Remove Data** symbol. Repeat for other time-lapse files.
    NOTE: Trajectory plots can be used to visualize the directness of individual cell paths for a given cell condition or state over the course of time-lapse movies (Figure 4A). Distance and speed data stored in the .txt files can then be used for further analysis.
3. **Quantification of neural crest cell area and circularity**
  1. Open the time-lapse .stk files in ImageJ and calibrate according to microscope settings, working in pixel/μm.
  2. Under **Analyze | Set Measurements**, click to select the cell shape parameters: cell area, perimeter and shape descriptor.
  3. Use the **Freehand Selection** tool to manually draw around each cell, using cell membrane boundaries as a guide.
  4. Press **Ctrl + B** keys on the keyboard to maintain the cell outline overlain on the image. Repeat for cells over each time-lapse frame.
  5. Use the **Image | Overlay | To ROI Manager** to store the values.
  6. Once all cells of interest per frame have been outlined, click **Measure**. Save the results as a .csv file.
    NOTE: 10–20 cells per movie should be outlined, with a total of 30–60 cells analyzed per condition (n = 3). Cell shape data (.csv files) can be used to quantify how cell shape dynamics change over time (Figure 4C) or how morphology may be altered under different cell treatments.

## Representative Results

Using the procedure demonstrated here, mouse embryos were dissected from the uterus, and extraembryonic tissues were removed (Figure 1A–D). Embryos were somite staged (using only embryos at 5–8 somites (ss), Figure 1E,F). The cranial neural plate was then dissected and the neuroepithelium was isolated. Mesodermal cells, identified as loose, circular, mesenchymal cells, were gently brushed off (Figure 1G-L). The anterior neural plate can be explanted whole, in which case the neural crest tissue will emerge laterally and expand radially around the explant, or each neural plate border (right and left) can be explanted separately. This is particularly useful when explanting from genetic mutants.

Within 24 h, a region of premigratory (epithelial) cranial neural crest can clearly be seen surrounding the neural plate explant (Figure 2B). Furthermore, a subpopulation of neural crest cells have undergone epithelial to mesenchymal transition and appear fully mesenchymal (Figure 2). Thus, we have several concentric rings of distinct cells, with the neural plate (NP) in the center, the premigratory neural crest (pNC) in the intermediate circle, and a population of migratory neural crest (mNC) in the outside ring (Figure 2B). In order to trace NC cells, it is possible to use genetically modified mouse models as we show in Figure 2C. In this case, we have used the neural crest specific *Wnt1::Cre;Rosa^mTmG^* which results in NC cells being labeled in green. In these mice, cells express membrane tomato (mT, in red) unless they are expressing Cre recombinase. Recombination leads to cells expressing membrane green fluorescent protein (GFP, in green). The red cells shown at the center of the explant are neural plate cells. Some dorsal neural plate cells also express GFP; for long term culture, we would excise all of the cells in the center. For our purposes, the purity of the explant is sufficient to track the different neural crest cell populations. Where higher purity of the neural crest is necessary, this genetic labeling strategy can be combined with fluorescent activated cell sorting (FACS) to ensure purity of the population. Alternatively, it is possible to fix the explants and identify the NC population with antibody labeling.

**Figure 3:**
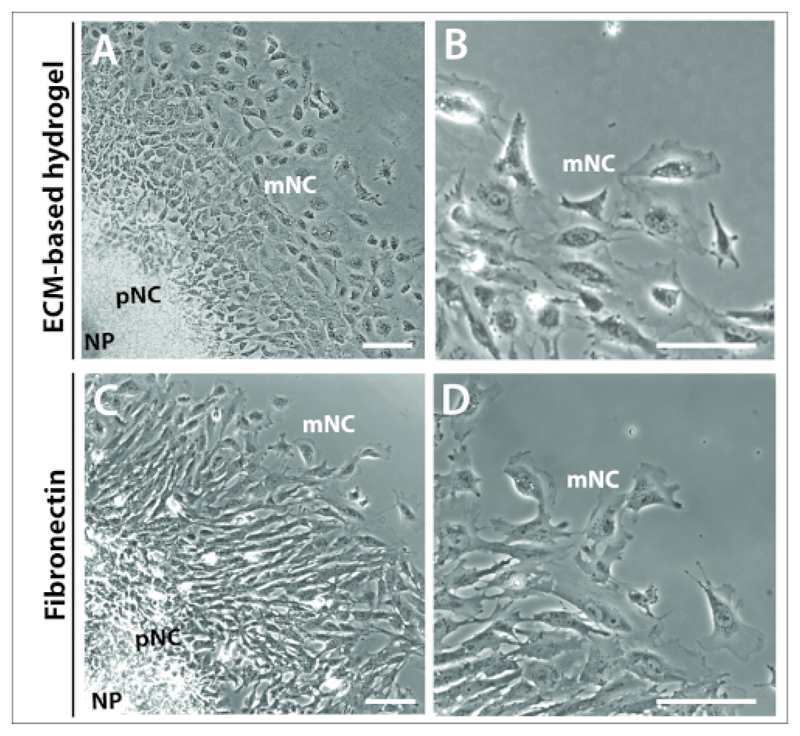
Explants cultured on different substrates. **(A–B)** Phase contrast images of explants cultured on commercial ECM-hydrogel. **(C–D)** Explants cultured on 1 μg/mL fibronectin. Neural plate (NP), premigratory (pNC) and migratory (mNC) neural crest cells can be distinguished by their differing cell morphologies. Scale bar = 100 μm.

It was also evident by 24 h that the characteristic concentric rings of premigratory and fully migratory NC cells of the explant cultures was not dependent nor governed by matrix choice (Figure 3). Explant cultures plated on both an ECM-based hydrogel and fibronectin formed comparable explant structures, comprising the three cell populations, NP, pNC and mNC (Figure 3A,C). Neural crest cell morphology was also comparable between those plated on the ECM-based hydrogel and fibronectin (Figure 3B,D). However, explants plated on fibronectin produced cells with more prominent lamellipodia at the cell leading edge, seemingly more polarized in the direction of migration (Figure 3B,D).

**Figure 4:**
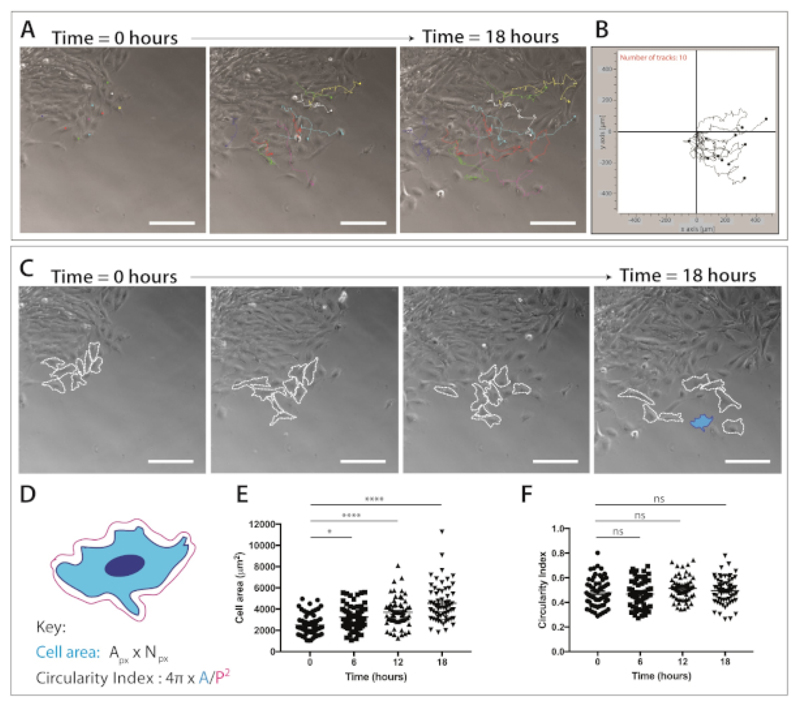
Quantification of cranial neural crest cell migration and cell shape dynamics. **(A)** Phase-contrast frames from time-lapse imaging of explant cultures overlaid with single neural crest cell tracks, using the ImageJ/Fiji Manual Cell Tracking plug-in. Ten representative mNC cells were manually tracked over 18 h (217 frames) and the XY coordinates were exported. Data are represented as an overlay dot and line plots. Cells were plated on 1 μg/mL fibronectin. Scale bar = 200 μm. **(B)** Representative trajectory plot of 10 mNC cells, generated using migration software. **(C)** Phase-contrast frames taken from time-lapse analysis of explant cultures. Dashed lines outline 8 representative migratory neural crest cells analyzed for cell shape dynamics when plated on 1 μg/mL fibronectin. Scale bar = 200 μm. **(D)** Schematic representation of the calculations used to quantify cell area and circularity. Cell morphology of the schematic is that of the cell highlighted in blue **(C)**. A_px_ = pixel area, N_px_ = pixel number, A = area, P = perimeter (1 pixel = 1.60772 μm^2^). **(E–F)** Quantification of cell area and cell circularity measures over time. Data represents mean ± SEM. Each dot represents one cell (n = 60), taken from 3 independent experiments, and analyzed at 0 h, 6 h, 12 h and 18 h (ns non-significant, * p<0.05, **** p < 0.0001 one-way ANOVA, Tukey’s multiple comparisons test).

Once a population of migratory neural crest cells is evident, live cell imaging can be completed. Time-lapse microscopy is set to 10x magnification (18 h, 1 frame/5 min) for subsequent analysis of NC cell migration (Figure 4A). ImageJ Manual Tracking plug-in generates XY coordinates of individual cells over all frames of the time-lapse movies (Figure 4B). These coordinates can be processed using the migration software. This software enables visualization of individual cell tracks over time (Figure 4B) and can be used to quantify accumulated and Euclidean distance, as well as cell speed.

Time-lapse imaging data also provides a wealth of information on cell morphology during the migration of cranial neural crest cells (Figure 4C). By outlining individual cell membranes, cell area and perimeter measurements can be calculated from all frames of the movies (Figure 4C). These measurements allow for the subsequent quantification of cell area and circularity (Figure 4D). Figure 4C shows an analysis of cell shape changes over 18 h. Note that as cells migrate away from the explant, cell area significantly increases while cell circularity remains relatively constant (one-way ANOVA, Tukey’s multiple comparisons test) (Figure 4E,F). This suggests that as cells depart from the epithelial edge and lose cell-cell contacts, they show increased cell spread area. Cell circularity measures did not significantly change over time; however, short-term changes in circularity may be seen if an increased number of time points are quantified. Cell circularity measures can also provide interesting data on cell shape dynamics in the presence of a chemotactic cue or under confined conditions.

## Discussion

Studying mammalian neural crest cells has been a challenge for scientists because of the in utero nature of mammalian development. *In vivo* studies are difficult to set up, as the embryo must be manipulated under conditions that mimic life in the uterus. In practice, it is nearly impossible to reproducibly culture these (E8+) embryos for longer than 24 h, especially for live imaging. Furthermore, neural crest induction and migration occur concurrently with neural tube closure and embryonic turning in the mouse; this is a crucial and stressful morphogenetic event, which frequently fails when embryos are cultured *ex utero*. Thus, the success rate of *ex utero* approaches is generally low. The use of immortalized NC cells^21^ is a useful tool to reduce animal use and it may provide a better source of neural crest cells for long-term analysis, transfection, and enrichment studies. However, there is clearly a need to reliably culture primary neural crest cells. Our method is applicable to mouse knock-out or conditional genetic models. A comparable method to ours has been described for other neural crest populations^20^; however, our method thoroughly describes the step-by-step isolation of murine cranial NC cells. We also describe the use of different matrices as well as the migration analysis procedure in detail.

To achieve consistent results, we found that special attention paid towards staging during the selection of embryos. Not surprisingly, the number of somites correlates with different stages in cranial NC development. Therefore, the knowledge of the embryo anatomy is very important before acquiring any experimental data. This approach can then be adapted towards isolating discrete populations of neural crest cells, depending on the biological question and the target cells.

Once the embryos are selected and dissected, mesodermal cells can be readily distinguished and should be removed to allow better visualization and to reduce contamination. For longer-term cultures, the neural plate tissue can be removed at 24 h of plating in order to prevent contamination by neural tissues. A further refinement could be the use of fluorescent lineage labeling (for example, using a *Wnt1::cre* or *Sox10::creERT* drivers combined with fluorescent reporters^24,25^) to distinguish neural crest cells from other tissues as shown in Figure 2C.

Previous reports have highlighted the potential of plating mouse NC explant cultures on different matrices, most commonly on commercial ECM hydrogels, fibronectin and collagen I^20,21,26^. In our hands, mouse cranial NC explant cultures are successfully grown on all three matrices, at concentrations specified in original reports (data not shown). The initial refined approach we adapted for our NC explant cultures used a commercial hydrogel as the matrix of choice, which primarily consisted of laminin and collagens^21^ (Figure 3A–B). However, composition of this hydrogel is not clearly defined, with unknown growth factor and protein content. As such, we have since shifted our approach to plating mouse NC explant cultures on fibronectin (Figure 3C–D). Fibronectin is well defined and highly expressed in the ECM and basement membranes along which NC cells migrate *in vivo*^28,29,30^. To optimize a fibronectin matrix that best replicates neural crest cell migration and morphology as seen using the hydrogel, we compared NC cell behaviors exhibited on the hydrogel against a titration of 0.25–30 μg/mL fibronectin, and defined 1 μg/mL fibronectin as providing ideal properties (data not shown). We believe that this preliminary work may help establish a framework for the systematic comparison of matrices, such as fibronectin, against those previously described, namely collagen and laminin^32,33,34^. It would be especially interesting to compare mouse NC cell migratory capacity on fibronectin versus collagen I, given that collagen-IA1 is endogenously secreted by mouse, avian and human NC cells^28,30,31,32^. Collagen I is therefore as relevant as fibronectin in the consideration of matrix choice. It is also worth acknowledging that the bioavailability of growth factors in the media may be altered by different matrix components, especially given the high serum content of our media. To overcome this, we are currently working to produce serum-free defined culture conditions. These defined media are successfully used in neural crest induction protocols in the pluripotent stem cell field, but require further optimization for our NC explant culture system^33,34^. Our work may also serve as a starting point for refining conditions for other types of NC cells such as cardiac and trunk NC, and for subsequent studies of NC differentiation. Most importantly, this protocol allows the isolation of cranial NC cells for a variety of applications. We envision studies on directed migration, 3-D migration and invasion. Cells isolated in this manner can be treated *in vitro* for a number of analyses. For example, cells can readily be treated using different small molecules to target specific proteins, they can be treated at defined time points, and washout experiments can be designed to determine recovery of cell behaviors (Figure 4). Longer term culture for transfection and differentiation assays is possible, as well as passage of cells (data not shown). However, the viability, the capacity of cell renewal and multipotency should be validated after passaging. Cells plated on glass coverslips can also be used in immunofluorescent staining protocols, following live imaging. Finally, this approach represents a tremendously powerful system for studying migration of NC from genetic mouse models^22,23,24,25^.
